# Medical History and Biography

**Published:** 1887-03

**Authors:** 


### MEDICAL HISTORY AND BIOGRAPHY.

The attempts to secure a complete and satisfactory history of the physicians of the United States have been noted with satisfaction, and should receive substantial recognition by every member of the profession.

While there are one or two valuable histories of this character, yet on account of the many difficulties which surround the making up of a general work on the subject, it has been impossible to render them as complete as they should be. To thoroughly cover the ground therefore, the publication of special works which treat of but a limited amount of territory seems to be demanded.

The medical history of single States therefore, will be found of particular interest to the general reader as well as to physicians, and invariably supplies an urgent want, as there is, as a rule, very little to be found concerning the pioneers and older members of the profession. There are one or two such treatises, we believe, already published. A medical history of Connecticut is already in press, and a similar work on New Hampshire is in preparation by Dr. Watson, the efficient Secretary of the State Board of Health, which will, no doubt, be read with interest and satisfaction by a large circle of professional and other readers.

There is no reason why every State which has any early history whatever, should not contribute its share toward an exhaustive history of the medical men of this country. There is certainly no class of men whose early struggles, hardships and devotion to the cause of science entitles them to a more lofty and enduring monument than the physicians of America, and every contribution to the history of such as these, should be and will be received with due appreciation.—*N. E. Medical Journal.*

The above from an able contemporary is reproduced with pleasure. In this connection we wish to call the attention of Texas physicians to the fact that the subscription list for biography of contemporary physicians of Texas is still open, and Mr. S. H. Dixon, who has the contract for finishing the canvass and for publishing the work, informs us that he will speedily push it to completion. A few more portraits only will be taken, and arrangements have been made to produce them at a lower figure than was at first announced. Address S. H. Dixon & Co., Austin, Texas.

